# Association of Vision Impairment With Cognitive Decline Across Multiple Domains in Older Adults

**DOI:** 10.1001/jamanetworkopen.2021.17416

**Published:** 2021-07-16

**Authors:** Varshini Varadaraj, Beatriz Munoz, Jennifer A. Deal, Yang An, Marilyn S. Albert, Susan M. Resnick, Luigi Ferrucci, Bonnielin K. Swenor

**Affiliations:** 1 Johns Hopkins Wilmer Eye Institute, Johns Hopkins University School of Medicine, Baltimore, Maryland; 2 Center for Disability Health Research, Johns Hopkins University, Baltimore, Maryland; 3 Department of Epidemiology, Johns Hopkins Bloomberg School of Public Health, Baltimore, Maryland; 4 Laboratory of Behavioral Neuroscience, Intramural Research Program, National Institute on Aging, Baltimore, Maryland; 5 Department of Neurology, Johns Hopkins University School of Medicine, Baltimore, Maryland; 6 Translational Gerontology Branch, National Institute on Aging, Baltimore, Maryland

## Abstract

**Importance:**

Associations between visual and global cognitive impairments have been previously documented, but there is limited research examining these associations between multiple measures of vision across cognitive domains.

**Objective:**

To examine the association between vision and cognitive across multiple cognitive domains using multiple measures of vision.

**Design, Setting, and Participants:**

This longitudinal cohort study used data from the Baltimore Longitudinal Study of Aging for 2003 to 2019. Participants in the current study were aged 60 to 94 years with vision and cognitive measures. Data analysis was performed from May 2020 to May 2021.

**Main Outcomes and Measures:**

Cognitive function was measured across multiple domains, including language, memory, attention, executive function, and visuospatial ability. Cognitive domain scores were calculated as the mean of standardized cognitive test scores within each domain. Visual function was assessed using measures of visual acuity, contrast sensitivity, and stereo acuity at baseline.

**Results:**

Analyses included 1202 participants (610 women [50.8%]; 853 White participants [71.0%]) with a mean (SD) age of 71.1 (8.6) years who were followed up for a mean (SD) of 6.9 (4.7) years. Worse visual acuity (per 0.1 logarithm of the minimal angle of resolution) at baseline was associated with greater declines in language (β, −0.0035; 95% CI, −0.007 to −0.001) and memory (β, −0.0052; 95% CI, −0.010 to −0.001) domain scores. Worse contrast sensitivity (per 0.1 log units) at baseline was associated with greater declines in language (β, −0.010; 95% CI, −0.014 to −0.006), memory (β, −0.009; 95% CI, −0.015 to −0.003), attention (β, −0.010; 95% CI, −0.017 to −0.003), and visuospatial ability (β, −0.010; 95% CI, −0.017 to −0.002) domain scores. Over the follow-up period, declines on tests of language (β, −0.019; 95% CI, −0.034 to −0.005) and memory (β, −0.032; 95% CI, −0.051 to −0.012) were significantly greater for participants with impaired stereo acuity compared with those without such impairment.

**Conclusions and Relevance:**

These findings suggest that the association between vision and cognition differs between visual acuity, contrast sensitivity, and stereo acuity and that patterns of cognitive decline may differ by type of vision impairment, with impaired contrast sensitivity being associated with declines across more cognitive domains than other measures of visual functioning.

## Key Points

**Question:**

Are there associations between visual and cognitive impairment across multiple cognitive domains and multiple measures of vision?

**Findings:**

In this longitudinal cohort study of 1202 older adults, worse visual acuity and stereo acuity impairment were associated with greater declines in language and memory domain scores, whereas worse contrast sensitivity was associated with declines in language, memory, attention, and visuospatial ability domain scores.

**Meaning:**

These findings suggest that the association between vision and cognition differs by measure of vision and that impaired contrast sensitivity is associated with declines across more cognitive domains than visual acuity.

## Teaser

This cohort study examines the association between visual and cognitive impairments across multiple cognitive domains using multiple measures of vision.

## Introduction

Previous research^1,2,3,4^ has demonstrated that older adults with vision impairment are at greater risk of cognitive decline. Multiple mechanisms have been proposed to explain this association, including common pathophysiological processes (eg, inflammation or atherosclerotic disease contributing to visual and cognitive declines)^4,5^ and/or that vision impairment is a factor associated with increased risk of cognitive decline via downstream consequences, such as decreased social interaction and physical activity.^1,3,6^

A recent systematic review and metanalysis^7^ reported that vision impairment is associated with 2.4-fold greater odds of cognitive impairment in existing cross-sectional studies and 1.7-fold greater odds in longitudinal studies. However, the association of vision impairment with specific cognitive domains has not been fully elucidated^8^; there is a lack of data and consensus in the literature on aging on whether the decline is global, process specific, domain specific, or specific to modality of test administration. A metanalysis^9^ examining the association between vision and 4 cognitive domains—attention, executive function, memory, and language—found that vision did not mediate age differences in these functioning domains. However, as is the case with many other studies,^2,10,11,12^ the results were based on a single measure of visual functioning—visual acuity—although vision is a complex process and more than 1 measure is needed to fully characterize vision loss. Moreover, there is limited longitudinal research examining the association between visual and cognitive function, which hinders our ability to determine the temporality of the vision-cognition relationship.^10,13^

This cohort study sought to build on prior work by using longitudinal data and multiple measures of both visual function and cognition to characterize the association between vision and cognitive domains in older adults. Using data from the Baltimore Longitudinal Study of Aging (BLSA), including 3 measures of visual function (visual acuity, contrast sensitivity, and stereo acuity) and 5 cognitive domains (language, memory, attention, executive function, and visuospatial ability), we tested our hypotheses that the association with cognitive domain decline differs by type of vision impairment. In addition, we hypothesized that worse visual function is associated with greater declines in cognitive domain scores on tests of language and memory.^14,15^

## Methods

### Study Population

The BLSA was established in 1958 and is a longitudinal study of physical and cognitive aging. Informed consent was obtained from all participants, and the BLSA protocol has been approved by the institutional review board of the National Institute of Environmental Health Science, National Institutes of Health. Only deidentified BLSA data were shared with us for the current analysis; thus, the Johns Hopkins Medicine institutional review board determined this study to be exempt. This study follows the Strengthening the Reporting of Observational Studies in Epidemiology (STROBE) reporting guideline.

Participants included community-dwelling volunteers in Baltimore, Maryland, who passed comprehensive health and functional screening evaluations at enrollment. The current study included adults aged 60 years and older who underwent vision and cognitive testing between 2003 and 2019. Participants underwent testing every 1 to 4 years depending on age (aged <60 years, every 4 years; aged 60-79 years, every 2 years; aged ≥80 years, every year), and therefore have different numbers of visits. Because the BLSA enrolls continuously, participants also have variable start times (ie, differential baseline visits) and follow-up times. More details on the study and enrollment procedures have been published elsewhere.^16,17^ The first visit that collected vision data served as the baseline visit for this analysis.

### Outcomes

The primary outcome of interest was cognition, measured by cognitive domain scores. A comprehensive BLSA cognitive battery measures cognitive function in multiple domains. We present data on 11 cognitive measures that assessed 5 cognitive domains, with each domain comprising 2 or more cognitive measures, as detailed in the subsequent section.

#### Cognitive Domains: Language, Memory, Attention, Executive Function, and Visuospatial Ability

Language was assessed using Verbal Fluency–Letters,^18^ Verbal Fluency–Categories,^19^ and the Boston Naming Test.^20^ Memory was assessed using immediate (sum of 5 trials) and long-delay free recall from the California Verbal Learning Test.^21^ Attention was assessed using Trail Making Test Part A^22,23^ and the Digit Span Forward subtest of the Wechsler Adult Intelligence Scale–Revised.^24^ Executive function was assessed using the Digit Span Backward subtest of the Wechsler Adult Intelligence Scale–Revised^24^ and a difference score representing the difference in time to complete (in seconds) trail B compared with trail A (Delta Trail Making Test).^23,25,26^ Visuospatial ability was assessed using the Card Rotations Test^27^ and 2 Clock Drawing Tests (CDTs),^28^ where participants were asked to draw the hands and face of a clock indicating 3:35 and 11:10. The mean of the 2 clock drawing tests was calculated, and the domain scores of visuospatial ability were computed using the mean of the standardized *z* scores from the Card Rotations Test and the mean of the CDTs.

To obtain domain scores, scores from individual cognitive tests were standardized (converted to a *z* score using the baseline mean and SD), and these *z* scores were then averaged within each cognitive domain. This approach, the cognitive domains, and the individual test components in each domain were chosen on the basis of previous work in BLSA,^29,30,31^ and the tests have been previously described in detail.^32,33^

#### Vision Measures: Visual Acuity, Contrast Sensitivity, and Stereo Acuity

The primary independent variables were 3 measures of visual function—visual acuity, contrast sensitivity, and visual fields—which were collected at the baseline visit. Visual acuity refers to how much a pattern must differ in size to be seen. From 2003 to 2014, visual acuity was measured binocularly with the participant’s presenting corrective lenses using the CSV-1000 eye chart (VectorVision) at an 8-foot (2.4 m) distance.^34^ The CSV-1000 tests 3 contrast levels of logarithm of the minimal angle of resolution (logMAR) acuity and contrast sensitivity with 1 instrument rather than 2. The BLSA vision protocol was updated in January 2015, after which visual acuity was measured unilaterally with the participant’s presenting corrective lenses using Early Treatment of Diabetic Retinopathy Study eye charts (Precision Vision) at a 3-m distance. Better-eye visual acuity was used for analysis under the updated protocol. Visual acuity data collected under the 2 different protocols were harmonized and equated using sliding windows and weighted polynomial regression. Visual acuity was calculated as the logMAR score and ranges from 0.80 to −0.30 logMAR, where lower values indicate better acuity. For analysis, visual acuity was treated as a continuous variable (per 0.1 worsening in logMAR).

Contrast sensitivity refers to how much a pattern must vary in contrast (ie, brightness) to be seen. From 2003 to 2014, under the initial BLSA protocol, contrast sensitivity was measured binocularly with participant’s presenting corrective lenses using the CSV-1000 eye chart at an 8-foot (2.4-m) distance. Under the updated BLSA protocol (2015-2019), a Pelli-Robson^35^ chart (Clement Clarke International) at a 1-m distance was used to measure contrast sensitivity bilaterally with the participant’s presenting corrective lenses. Similar to visual acuity, contrast sensitivity collected under the 2 different protocols were harmonized and equated using sliding windows and weighted regression. Contrast sensitivity was calculated as log contrast units, which indicates the lowest contrast threshold discerned, and ranges from 0 to 2.25 log contrast units, where higher values indicate better contrast sensitivity. For analysis, contrast sensitivity was treated as a continuous variable (per 0.1 worsening in log contrast units).

Stereo acuity, which is a measure of depth perception, was measured with the Stereo Fly Test Circles pattern (SO-001) instrument (Stereo Optical). It was tested bilaterally at a 16-inch (41-cm) distance using recommended special 3-dimensional glasses for testing worn over the participant’s presenting corrective lenses for near vision. Participants were presented with stereo images of decreasing depth differentials over 9 trials (ranging from 800 to 40 seconds of arc), and the smallest depth disparity that was correctly discerned was recorded as the stereo acuity value. There was no change in protocol for stereo acuity across years. For analysis, stereo acuity impairment was defined as the inability to ascertain a depth differential of 60 seconds of arc or smaller.

### Other Covariates

Information on age, sex, race (White, Black, or other, which included American Indian or Alaska Native, Chinese, Filipino, Japanese, other Asian or Pacific Islander, other non-White, and not classifiable), educational attainment (in years, categorized as high school or less, any college, and graduate school), and smoking status (current, former, or never), were collected via self-report and recorded from the baseline visit. Race was analyzed in this study because it may be a potential confounder of the associations between vision function and cognitive domain scores. A diagnosis of diabetes was based on a fasting glucose level of more than 125 mg/dL (to convert to millimoles per liter, multiply by 0.0555), a pathological oral glucose tolerance test result (plasma glucose ≥200 mg/dL at 2 hours), or self-report of a physician diagnosis plus treatment with oral antidiabetic drugs or insulin. The diagnosis of hypertension was based on a systolic blood pressure of greater than 140 mm Hg, and/or diastolic blood pressure of at least 90 mm Hg, or self-report of a physician diagnosis plus treatment with antihypertensive medications.

### Statistical Analysis

Demographic, health, and vision measures were summarized using means and SDs for continuous variables and frequency counts and percentages for categorical variables. Linear mixed models were used to examine the cross-sectional association between vision and cognitive performance. Linear mixed models with random intercept and slope were used to examine the longitudinal associations between the vision variables at baseline and annual rates of change in mean *z* scores for each of the 5 cognitive domains. In longitudinal analyses, vision variables were treated as baseline time-fixed factors associated with the cognitive outcomes, and all models were adjusted for age (continuous), sex, race, education, smoking status, hypertension, and diabetes. Visual acuity and contrast sensitivity measures were examined on a continuous scale to assess change in cognitive domain scores for each unit change in visual function. Stereo acuity was dichotomized as impaired or not impaired according to a clinically meaningful cut point because it not a continuous measure.

We conducted the following sensitivity analyses. First, we explored adding a spline term for age for participants aged 80 years (guided by the visualization of the baseline cross-sectional associations of age and cognitive domain scores) to allow for a different age effect for older participants. Second, the BLSA cognitive battery includes both visual and nonvisual tests. To determine the association of vision with tests of cognition that incorporated items presented visually, we conducted sensitivity analyses in which the visual cognitive tests were excluded (indicated in eTable 1 in the Supplement), in the calculation of the domain scores to ensure that any change in cognitive domain scores noted reflect visual rather than cognitive impairments. These analyses therefore excluded the visuospatial ability domain in its entirety. Third, we conducted sensitivity analysis stratifying by time period (ie, before [1098 participants] and after [134 participants] January 2015) to examine whether the change in assessing acuity and contrast sensitivity test was associated with the outcomes. Finally, we ran regression models to assess the association between any vision impairment (ie, visual acuity [>0.3 logMAR], contrast sensitivity [<1.7 log contrast], or stereo acuity impairment vs no vision impairment) and annual changes in cognitive domain scores. The dichotomized visual acuity and contrast sensitivity impairments included in this composite measure were defined according to clinically meaningful cut points. Fourth, we also conducted sensitivity analysis excluding the 188 participants with only baseline visits from all longitudinal models (indicated in eTable 2 and eTable 3 in the Supplement). Fifth, we conducted sensitivity analysis including a comorbidity index that was calculated using the sum of indicator variables for the presence of the following health issues: (1) self-reported heart disease or cardiac surgery (includes myocardial infraction, congestive heart failure, angina, coronary artery bypass surgery, and angioplasty); (2) exertional chest pain, calf pain, or shortness of breath; (3) diabetes (currently receiving treatment); (4) pulmonary disease; (5) cerebral vascular disease (stroke or transient ischemic attack); and (6) self-reported diagnosis of lower extremity arthritis. Of note, diabetes and hypertension were not adjusted for separately here, as done in the primary models.

Missing data were minimal and, thus, were treated as missing at random. Statistical significance was defined at *P* < .05, and T-type confidence limits and 2-sided *P* values are presented. All statistical analyses were performed using SAS statistical software version 9.1 (SAS Institute). Data analysis was performed from May 2020 to May 2021.

## Results

### Population Characteristics

Of the 1234 participants initially enrolled, 32 participants with mild cognitive impairment or dementia with onset of symptoms at the baseline examination visit or before were excluded, leaving a final analytical sample of 1202 cognitively normal older adults with 5256 study visits (Figure). The participants had a mean (SD) age of 71.1 (8.6) years and were followed up for a mean (SD) of 6.9 (4.7) years (Table 1). The follow-up time ranged from 0 (188 participants only had a baseline visit) to 16.8 years (median [interquartile range], 6.4 [3.0-10.9] years). The majority of participants (853 participants [71.0%]) attended 3 or more study visits from 2003 to 2019. Approximately one-half of the participants were female (610 women [50.8%]), and the majority were White (853 participants [71.0%]), had a graduate school degree (761 participants [63.3%]), did not smoke (1152 participants [96.1%]), and did not have diabetes (966 participants [80.4%]) or hypertension (761 participants [63.3%]). At baseline, participants had a mean (SD) visual acuity of 0.16 (0.16) logMAR and contrast sensitivity of 1.90 (0.12) log units, and 14.5% (172 participants) had stereo acuity impairment. Smoking status was missing for 3 participants (0.2%), contrast sensitivity measures were missing at baseline for 23 participants (1.9%), and stereo acuity was missing for 45 participants (3.7%).

**Figure.**
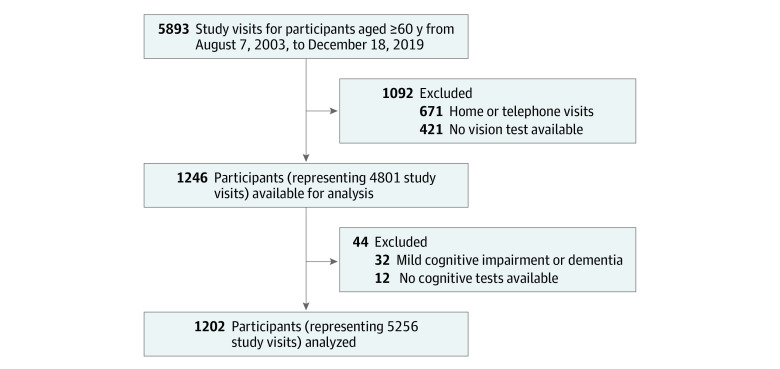
Study Participant Selection, Baltimore Longitudinal Study on Aging, 2003-2019

**Table 1.** Demographic and Clinical Characteristics of Participants at Baseline, Baltimore Longitudinal Study on Aging, 2003-2019

Characteristics	Participants, No. (%) (N = 1202)
Demographic	
Age, mean (SD) [range], y	71.1 (8.6) [60-94]
Age categories, y	
60-79	945 (78.6)
≥80	257 (21.4)
Sex	
Female	610 (50.8)
Male	592 (49.2)
Race	
White	853 (71.0)
Black	281 (23.4)
Other^a^	67 (5.6)
Education	
High school or less	69 (5.7)
At least some college, completed college	372 (31.0)
Graduate school	761 (63.3)
Clinical	
Visits, mean (SD), No. (N = 5256)	4.4 (2.7)
Follow-up time, mean (SD), y	6.9 (4.7)
Visits, No.	
1	188 (15.7)
2	160 (13.3)
≥3	853 (71.0)
Health measures	
Smoking	
Never or quit >10 y ago	1152 (96.1)
Current or quit <10 y	47 (3.9)
Body mass index^b^	
<25	431 (35.9)
25 to <30	486 (40.5)
≥30	284 (23.6)
Diabetes	236 (19.6)
Hypertension	441 (36.7)
Visual measures	
Visual acuity, mean (SD), logarithm of the minimum angle of resolution	0.16 (0.16)
Contrast sensitivity, mean (SD), log units^c^	1.91 (0.12)
Stereo acuity impairment (worse than 60 s of arc)^d^	161 (13.9)
Any vision impairment^e^	321 (27.4)

^a^ Other refers to American Indian or Alaska Native, Chinese, Filipino, Japanese, other Asian or Pacific Islander, other non-White, and not classifiable.

^b^ Body mass index is calculated as weight in kilograms divided by height in meters squared.

^c^ Data were missing for 23 participants.

^d^ Data were missing for 45 participants.

^e^ Refers to either visual acuity, contrast sensitivity, or stereo acuity impairment.

### Cross-sectional Associations

In fully adjusted cross-sectional analysis, worse visual acuity (per 0.1 logMAR) was associated with lower scores in the domains of language (β, −0.041; 95% CI, −0.067 to −0.015), attention (β, −0.052; 95% CI, −0.078 to −0.025), executive function (β, −0.046; 95% CI, −0.069 to −0.014), and visuospatial ability (β, −0.076; 95% CI, −0.106 to −0.045) (Table 2). Worse contrast sensitivity (per 0.1 log contrast units) was associated with lower scores in visuospatial ability only (β, −0.072; 95% CI, −0.113 to −0.031). Stereo acuity impairment was associated with lower scores on tests of executive function only (β, −0.142; 95% CI, −0.263 to −0.045).

**Table 2.** Standardized, Multivariable-Adjusted, Population Mean Estimates and Difference in Estimates of Annual Rates of Domain-Specific Cognitive Decline by Baseline Visual Acuity and Contrast Sensitivity, Baltimore Longitudinal Study on Aging, 2003-2019^a^

Visual measures and cognitive domains	β (95% CI)			*P* value
	Difference at baseline per 0.1 log unit worse visual acuity or contrast sensitivity	Annual rate of change for reference group (median visual acuity or contrast sensitivity at baseline)^b^	Difference (acceleration) in annual rate of change in the slope per 0.1 log unit worse baseline visual acuity or contrast sensitivity	
Visual acuity				
Language	−0.041 (−0.067 to −0.015)^c^	−0.040 (−0.045 to −0.035)^c^	−0.0035 (−0.007 to −0.001)^c^	.007^c^
Memory	−0.022 (−0.054 to 0.010)	−0.031 (−0.038 to −0.025)^c^	−0.0052 (−0.010 to −0.001)^c^	.02^c^
Attention	−0.052 (−0.078 to −0.025)^c^	−0.065 (−0.073 to −0.056)^c^	−0.0041 (−0.009 to 0.001)	.11
Executive function	−0.041 (−0.069 to −0.014)^c^	−0.036 (−0.041 to −0.025)^c^	−0.0039 (−0.008 to 0.000)	.05
Visuospatial ability	−0.076 (−0.106 to −0.045)^c^	−0.048 (−0.057 to −0.040)^c^	−0.001 (−0.006 to 0.004)	.74
Contrast sensitivity				
Language	−0.027 (−0.062 to 0.009)	−0.037 (−0.043 to −0.032)^c^	−0.010 (−0.014 to −0.006)^c^	<.001^c^
Memory	−0.041 (−0.085 to 0.002)	−0.030 (−0.037 to −0.023)^c^	−0.009 (−0.015 to −0.003)^c^	.004^c^
Attention	−0.017 (−0.054 to 0.020)	−0.063 (−0.072 to −0.055)^c^	−0.010 (−0.017 to −0.003)^c^	.004^c^
Executive function	−0.037 (−0.075 to 0.000)	−0.035 (−0.041 to −0.029)^c^	−0.005 (−0.011 to 0.0003)	.07
Visuospatial ability	−0.072 (−0.113 to −0.031)^c^	−0.045 (−0.053 to −0.036)^c^	−0.010 (−0.017 to −0.002)^c^	.01^c^

^a^ All models were adjusted for baseline visual acuity or contrast sensitivity, age, sex, race, education, smoking status, hypertension, and diabetes.

^b^ Refers to participants with 0.1 logarithm of the minimum angle of resolution (median at baseline) in visual acuity models and participants with 1.95 log contrast (median at baseline) for contrast sensitivity models.

^c^ Statistically significant at *P* < .05.

### Longitudinal Associations

In fully adjusted longitudinal regression analysis, worse visual acuity (per 0.1 logMAR) at baseline was associated with greater annual declines in language (β, −0.0035; 95% CI, −0.007 to −0.001) and memory (β, −0.0052; 95% CI, −0.010 to −0.001) domain scores (Table 2). Worse contrast sensitivity (per 0.1 log contrast units) at baseline was associated with greater declines in language (β, −0.010; 95% CI, −0.014 to −0.006), memory (β, −0.009; 95% CI, −0.015 to −0.003), attention (β, −0.010; 95% CI, −0.017 to −0.003), and visuospatial ability (β, −0.010; 95% CI, 0.017 to −0.002) domain scores. Compared with older adults without stereo acuity impairment at baseline, older adults with stereo acuity impairment had greater declines in language (β, −0.019; 95% CI, −0.034 to −0.005) and memory (β, −0.032; 95% CI, −0.051 to −0.012) (Table 3).

**Table 3.** Standardized, Multivariable-Adjusted, Population Mean Estimates and Difference in Estimates of Annual Rates of Domain-Specific Cognitive Decline by Baseline Stereo Acuity Impairment, Baltimore Longitudinal Study on Aging, 2003-2019^a^

Cognitive domain	β (95% CI)			*P* value
	Difference at baseline between the stereo acuity non-impaired and impaired	Annual rate of change for reference group (no stereo acuity impairment at baseline)	Difference (acceleration) in annual rate of change in the slope for group with stereo acuity impairment at baseline	
Language	−0.042 (−0.157 to 0.073)	−0.039 (−0.045 to −0.034)^b^	−0.019 (−0.034 to −0.005)^b^	.008^b^
Memory	−0.060 (−0.203 to 0.082)	−0.028 (−0.035 to −0.021)^b^	−0.032 (−0.051 to −0.012)^b^	.001^b^
Attention	−0.033 (−0.154 to 0.087)	−0.063 (−0.072 to −0.055)^b^	−0.016 (−0.039 to 0.007)	.17
Executive function	−0.142 (−0.263 to −0.022)^b^	−0.036 (−0.042 to −0.030)^b^	−0.006 (−0.022 to 0.011)	.49
Visuospatial ability	−0.087 (−0.220 to 0.045)	−0.045 (−0.054 to −0.036)^b^	−0.022 (−0.046 to 0.003)	.08

^a^ All models were adjusted for baseline stereo impairment status, age, sex, race, education, smoking status, hypertension, and diabetes.

^b^ Statistically significant at *P* < .05.

### Sensitivity Analysis

In sensitivity analyses using a spline term at age 80 years, results were similar to those of the primary analyses (results not shown). In other sensitivity analyses including the nonvisual cognitive tests only (eTable 1 in the Supplement) in the calculation of the domain scores, inferences from our primary analyses were unchanged. In sensitivity analyses examining the association of any vision impairment (ie, visual acuity, contrast sensitivity, or stereo acuity impairment) with cognitive domain scores, greater declines were noted in language and memory, but not the other domains (results not shown). In analyses restricted to participants with more than 1 visit (ie, excluding the 188 participants with only baseline visits) from all longitudinal models, inferences from our primary analyses including the total population were unchanged (eTable 2 and eTable 3 in the Supplement). Compared with participants with 2 or more visits, participants who only had 1 visit during the study period had worse scores in memory and were more likely to be older and diabetic and less likely to be obese (eTable 4 in the Supplement). Finally, results were robust to sensitivity analysis including adjustment for a comorbidity index in all models (results not shown).

## Discussion

In this longitudinal cohort study of 1202 older adults, the association between vision and cognition differed across visual acuity, contrast sensitivity, and stereo acuity, indicating that patterns of domain-specific cognitive decline may differ by type of vision impairment. Furthermore, impaired contrast sensitivity was associated with declines across more cognitive domains than other measures of visual functioning, suggesting that impaired contrast sensitivity may be associated with greater cognitive decline than visual acuity, which is more commonly measured. Overall, these results add new support to the growing data reinforcing the hypothesis that vision impairment may be a factor associated with increased risk of cognitive decline or may reflect ongoing brain changes associated with cognitive processing.

These results are consistent with findings from prior studies that have documented an association between vision impairment and cognitive decline. Longitudinal data from both the Study of Osteoporotic Fractures^2^ and the Salisbury Eye Evaluation^3^ showed that visual acuity impairment was associated with cognitive decline. Our findings are also consistent with limited prior research demonstrating the association of vision measures beyond visual acuity with cognitive function in late life. For example, our prior work in the Health, Aging, and Body Composition Study^1^ showed that impaired contrast sensitivity and stereo acuity, in addition to visual acuity, were associated with cognitive declines and incident cognitive impairment over 9 years of follow-up. In addition, in the Study of Osteoporotic Fractures,^36^ a study of community-based older women, reduced contrast sensitivity (lowest quartile vs the highest) was associated with more than double the risk of incident mild cognitive impairment and dementia over 10 years of follow-up. The Epidemiology of Hearing Loss Study^4^ also showed that older adults with contrast sensitivity impairment were at increased risk of cognitive impairment over 10 years of follow-up. Taken together, this literature indicates that contrast sensitivity may be an important factor associated with cognitive decline, highlighting the need for future studies to assess multiple objective vision measures to more thoroughly capture the associations of visual impairment with cognition.

The majority of these existing studies, however, have relied on general cognitive performance such as the Mini-Mental State Examination (MMSE) to examine the association of vision impairment with cognitive decline.^1,2,3,4^ Therefore, there is a gap in knowledge about whether vision impairment is associated with general cognitive decline or, rather, with specific domains of cognitive impairment. An exception is the Maine-Syracuse Longitudinal Study,^8^ a community-based study of aging, that recently demonstrated that worse visual acuity was associated with 5-year declines, not in only global cognitive function but also specifically in visuospatial organization and memory and verbal episodic memory. In the current study, worse visual acuity was associated with greater declines in language and memory, as was worse contrast sensitivity and stereo acuity. These results support our hypothesis that worse visual function especially affects language and memory. Our results are also bolstered by evidence indicating that patients with age-related macular degeneration have lower scores on tests of memory and verbal fluency.^14,15^

A number of mechanisms have been hypothesized to explain these observed associations between visual functional and cognitive impairments.^37,38^ A shared neuropathological cause underlying both vision and cognitive impairments, such as microvascular disease, is a possibility.^39,40^ However, our findings were robust to adjustment for known confounders and factors commonly associated with the risk of vascular disease, such as diabetes, hypertension, and smoking. Alternatively, vision impairment may be associated with cognitive decline potentially through conditions known to affect cognition, such as depression, social isolation, loss of cognitively stimulating activities (eg, reading), and cognitive load (ie, the greater dedication of cognitive resources to visual processing may be detrimental to other cognitive processes).^1,2,3,6^ We hypothesize that a combination of the common cause and sensory loss consequence pathways likely contributes to cognitive decline in older adults with vision impairment.

Of note, many cognitive tests are vision dependent, and it can be argued that the worse cognitive domain scores in older adults with vision impairment may reflect visual rather than cognitive impairments. However, previous work in the BLSA^41^ using item response theory methods to test for differential item functioning by visual acuity impairment found no differential item functioning by visual impairment for cognitive tests that do not rely on vision, indicating that vision impairment does not affect completion of cognitive testing in this study population. Nevertheless, in another clinic-based sample of older adults,^42^ no differences in scores were noted between groups with and without vision impairment on vision-independent cognitive tests (verbal fluency, category fluency, and Rey Auditory Verbal Learning tests), but adults with vision impairments performed worse on the vision-dependent items of the MMSE and CDT, both of which are partly vision-dependent tests. However, when vision-independent items were used to replace the vision-dependent components of the MMSE and CDT, no group differences were noted, implying that vision impairment may contribute to worse performance. To address this potential issue, in sensitivity analysis, we excluded the vision-dependent tasks from each cognitive domain, and the results were unchanged. However, the impact of using vision-dependent cognitive tests in populations with vision impairment needs to be further evaluated, and perhaps novel vision-independent cognitive screening tests need to be developed for accurate diagnosis of cognitive decline for visually impaired older adults.

### Strengths and Limitations 

A strength of this study is the novel examination of the association of vision impairment with specific cognitive domains, whereas, to our knowledge, almost all prior work to date in this area has examined the association between vision impairment and global cognition. Although the results from this study suggest that worse visual function may affect some cognitive domains (ie, language and memory) more than others, future work needs to replicate these findings in other data sets. In addition, next steps to further elucidate the vision and cognition association needs to include neuroimaging studies to determine whether we see brain changes in the areas we would expect on the basis of these results. Ultimately, these results can be used to develop mitigation and intervention strategies for older adults with vision impairment.

This study also has limitations that should be considered when interpreting the results. First, the Digit Symbol Substitution Test (DSST), which is commonly used to assess executive function,^32,43^ was excluded from our analysis because the DSST was introduced into the BLSA neurocognitive battery in August 2005 and was, therefore, not collected at the baseline visit for our study population enrolled before then. However, in sensitivity analysis restricted to the population who underwent the DSST and for whom it was included in the executive function domain, inferences were unchanged (data not shown). Second, as discussed in the Methods section, the testing protocol for visual acuity and contrast sensitivity changed in 2015, which could affect the results of this longitudinal study. To account for this, we harmonized the vision data collected under the different protocols using robust statistical methods that equated the measurements scales and harmonized the data. To further examine the impact of this change in protocol, we conducted a sensitivity analysis stratifying by time period (ie, before and after January 2015) to examine whether the change in visual acuity and contrast sensitivity test was associated with the outcomes (data not shown). Overall, larger declines in domain estimates were noted with the original tests compared with the updated tests, but inferences were the same as those in our primary analyses, and these differences are likely due to the small sample size of BLSA participants with visits after the vision protocol change in 2015. Despite these measures taken, it is still possible that the change in vision testing protocols in 2015 contributed to the differences in our cross-sectional and longitudinal associations between vision measures and cognitive domain scores. Therefore, further longitudinal studies using uniform testing parameters over time are warranted. Third, compared with participants with 2 or more visits, participants who only had 1 visit during the study period had worse scores in memory and were more likely to be older and diabetic, and less likely to be obese. Nonetheless, our estimates are likely conservative because of this survivor bias–related attrition. Fourth, another limitation is reverse causation, because persons with cognitive impairment may not be able to participate fully or report accurately in assessments of visual function and may, thus, appear more visually impaired than they actually are. Fifth, we acknowledge that there may be potential survival bias and competing risks on cognitive decline, which may have biased our results in either positive or negative directions. However, these analyses were considered beyond the scope of this article. Sixth, our results may not be broadly generalizable because our study population consisted of predominantly White, well-educated, older adults. Subsequent studies should address these limitations by assessing vision at multiple time points and in broader study cohorts, allowing a more robust modeling of the longitudinal relationship between vision and cognitive outcomes.

## Conclusions

The findings of this cohort study indicate that among a population of community-dwelling, older adults without dementia, worse visual acuity, contrast sensitivity, and stereo acuity impairment are factors associated with increased risk of cognitive decline. Furthermore, the patterns of cognitive decline differ by type of vision measure, and the data suggest that contrast sensitivity impairment may affect cognitive decline across more domains than other measures of vision. These results add further evidence to the interrelationship between vision and eye health with healthy brain aging and highlight the need for research into the impact of vision and eye health interventions on cognitive outcomes.

## Notes

Supplement. **eTable 1.** Distribution of Cognitive Test Scores in Each Domain at the Baseline Visit, Baltimore Longitudinal Study on Aging, 2003-2019 **eTable 2.** Standardized, Multivariable‐Adjusted, Population‐Average-Estimates and Difference in Estimates of Annual Rates of Domain-Specific Cognitive Decline by Baseline Visual Acuity and Contrast Sensitivity, Baltimore Longitudinal Study on Aging (2003-2019), N=1014 Participants (Excluding 188 Participants With Only a Baseline Visit) **eTable 3.** Standardized, Multivariable‐Adjusted, Population‐Average-Estimates and Difference in Estimates of Annual Rates of Domain-Specific Cognitive Decline by Baseline Stere Acuity Impairment, Baltimore Longitudinal Study on Aging (2003-2019), N=1014 Participants (Excluding 188 Participants With Only a Baseline Visit) **eTable 4.** Demographic and Clinical Characteristics of Participants at Baseline, Baltimore Longitudinal Study on Aging (2003-2019), by Inclusion in the Longitudinal Sensitivity Analysis
