# Supplementary material for: Association of Vision Impairment With Cognitive Decline Across Multiple Domains in Older Adults
Source: JAMA Netw Open. 2021 Jul 16;4(7):e2117416. doi: 10.1001/jamanetworkopen.2021.17416 (PMC8285732; doi:10.1001/jamanetworkopen.2021.17416)
Supplement: Supplement. — eTable 1. Distribution of Cognitive Test Scores in Each Domain at the Baseline Visit, Baltimore Longitudinal Study on Aging, 2003-2019 eTable 2. Standardized, Multivariable‐Adjusted, Population‐Average-Estimates and Difference in Estimates of Annual Rates of Domain-Specific Cognitive Decline by Baseline Visual Acuity and Contrast Sensitivity, Baltimore Longitudinal Study on Aging (2003-2019), N=1014 Participants (Excluding 188 Participants With Only a Baseline Visit) eTable 3. Standardized, Multivariable‐Adjusted, Population‐Average-Estimates and Difference in Estimates of Annual Rates of Domain-Specific Cognitive Decline by Baseline Stere Acuity Impairment, Baltimore Longitudinal Study on Aging (2003-2019), N=1014 Participants (Excluding 188 Participants With Only a Baseline Visit) eTable 4. Demographic and Clinical Characteristics of Participants at Baseline, Baltimore Longitudinal Study on Aging (2003-2019), by Inclusion in the Longitudinal Sensitivity Analysis [file jamanetwopen-e2117416-s001.pdf]

Supplemental Online Content

Varadaraj V, Munoz B, Deal JA, et al. Association of vision impairment with cognitive decline across multiple domains in older adults. *JAMA Netw Open*. 2021;4(7):e2117416. doi:10.1001/jamanetworkopen.2021.17416

eTable 1. Distribution of Cognitive Test Scores in Each Domain at the Baseline Visit, Baltimore Longitudinal Study on Aging, 2003-2019
eTable 2. Standardized, Multivariable-Adjusted, Population-Average-Estimates and Difference in Estimates of Annual Rates of Domain-Specific Cognitive Decline by Baseline Visual Acuity and Contrast Sensitivity, Baltimore Longitudinal Study on Aging (2003-2019), N=1014 Participants (Excluding 188 Participants With Only a Baseline Visit)
eTable 3. Standardized, Multivariable-Adjusted, Population-Average-Estimates and Difference in Estimates of Annual Rates of Domain-Specific Cognitive Decline by Baseline Stere Acuity Impairment, Baltimore Longitudinal Study on Aging (2003-2019), N=1014 Participants (Excluding 188 Participants With Only a Baseline Visit)
eTable 4. Demographic and Clinical Characteristics of Participants at Baseline, Baltimore Longitudinal Study on Aging (2003-2019), by Inclusion in the Longitudinal Sensitivity Analysis

This supplemental material has been provided by the authors to give readers additional information about their work.

© 2021 Varadaraj V et al. *JAMA Network Open.*

eTable 1. Distribution of Cognitive Test Scores in Each Domain at the Baseline Visit, Baltimore Longitudinal Study on Aging, 2003-2019

Cognitive Tests per Domain	N	Mean (SD)	Median (IQR)	Min, Max
**Language**				
Verbal Fluency-Categories*	1183	15.8 (3.5)	15.7 (13.3, 18.0)	5.0, 32.0
Verbal Fluency-Letters*	1185	14.3 (4.1)	14.3 (11.3, 17.0)	1.7, 29.3
Boston Naming	1156	54.2 (6.0)	56 (52, 58)	16, 60
**Memory**				
CVLT: Immediate free recall*	1167	51.3 (12.0)	52 (42, 60)	17,80
CVLT: Long-delayed free recall*	1154	10.7 (3.4)	11 (8, 14)	0,16
**Attention**				
TMT Part A (log scale)	1180	1.49 (0.15)	1.48 (1.40, 1.58)	0.70, 2.28
WAIS-R Digit Span Forward*	1150	8.1 (2.3)	8 (6, 10)	2, 14
**Executive Function**				
WAIS-R Digit Span Backward*	1147	7.0 (2.2)	7 (6, 8)	0, 14
Delta TMT: Trail B – Trail A (in seconds)	1156	52.5 (37.6)	43 (29, 63)	-24, 275
**Visuospatial Ability**				
Clock-Drawing Test 3:25	1035	8.6 (1.0)	9 (8, 9)	3,10
Clock-Drawing Test 11:10	1162	9.1 (1.1)	9 (8, 10)	4, 10
Card Rotations test	1146	82.9 (38.8)	79 (55, 107)	-5, 204
**Domains (Global scores)**				
Language	1187	0.01 (0.74)	0.07 (-0.41, 0.49)	-3.28, 2.51
Memory	1167	-0.02 (0.95)	0.00 (-0.70, 0.74)	-2.69,1.93
Attention	1200	-0.01 (0.80)	-0.03 (-0.50,0.54)	-5.29, 3.48
Executive function	1197	0.00 (0.82)	0.07 (-0.42, 0.51)	-5.77, 2.27
Visuospatial ability	1193	-0.02 (0.87)	-0.06 (-0.61,0.53)	-2.82, 2.52

Abbreviations: CVLT= California Verbal Language Test, TMT= Trail Making Test, WAIS-R=Wechsler Adult Intelligence Scale—Revised
*Non-visual cognitive task

© 2021 Varadaraj V et al. *JAMA Network Open.*

eTable 2. Standardized, Multivariable-Adjusted, Population-Average-Estimates and Difference in Estimates of Annual Rates of Domain-Specific Cognitive Decline by Baseline Visual Acuity and Contrast Sensitivity, Baltimore Longitudinal Study on Aging (2003-2019), N=1014 Participants (Excluding 188 Participants With Only a Baseline Visit)

Cognitive Domain	Difference at baseline per 0.1 log unit worse visual acuity/contrast sensitivity	Annual rate of change for reference group (median visual acuity/contrast sensitivity at baseline)	Difference (acceleration) in annual rate of change in the slope per 0.1 log unit worse baseline visual acuity/contrast sensitivity
Visual Acuity			
	β (95% CI)	*β (95% CI)*[a]	*β (95% CI), p-value*
Language	**-0.045 (-0.073 to -0.017)**	**-0.040 (-0.045 to -0.035)**	**-0.0042 (-0.007 to -0.001), 0.008**
Memory	-0.024 (-0.059 to 0.010)	**-0.033 (-0.040 to -0.026)**	**-0.0051 (-0.010 to -0.001) 0.02**
Attention	**-0.049 (-0.077 to -0.021)**	**-0.066 (-0.074 to -0.058)**	-0.0045 (-0.010 to 0.001), 0.09
Executive function	**-0.037 (-0.066 to -0.008)**	**-0.036 (-0.042 to -0.030)**	**-0.0041 (-0.008 to -0.002), 0.04**
Visuospatial ability	**-0.074 (-0.107 to -0.041)**	**-0.049 (-0.058 to -0.041)**	-0.001 (-0.006 to 0.0045), 0.74
Contrast Sensitivity			
Language	-0.017 (-0.056 to 0.021)	**-0.038 (-0.043 to -0.033)**	**-0.010 (-0.015 to -0.006), <0. 001**
Memory	-0.031 (-0.078 to 0.015)	**-0.032 (-0.039 to -0.025)**	**-0.009 (-0.015 to -0.003), 0.003**
Attention	-0.002 (-0.041 to 0.036)	**-0.065 (-0.073 to -0.057)**	**-0.012 (-0.019 to -0.005), 0.001**
Executive function	-0.017 (-0.056 to 0.023)	**-0.036 (-0.042 to -0.030)**	**-0.006 (-0.012 to -0.0005), 0.03**
Visuospatial ability	**-0.053 (-0.098 to -0.009)**	**-0.046 (-0.054 to -0.037)**	**-0.010 (-0.018 to -0.003), 0.007**

All models adjusted for baseline visual acuity/contrast sensitivity, age, sex, race, education, smoking status, hypertension, and diabetes

[a] For a participant with 0.1 logMar (median at baseline) in visual acuity models, and for a participant with 1.95 log contrast (median at baseline) for contrast sensitivity models

Bold: P<0.05

© 2021 Varadaraj V et al. *JAMA Network Open.*

eTable 3. Standardized, Multivariable-Adjusted, Population-Average-Estimates and Difference in Estimates of Annual Rates of Domain-Specific Cognitive Decline by Baseline Stere Acuity Impairment, Baltimore Longitudinal Study on Aging (2003-2019), N=1014 Participants (Excluding 188 Participants With Only a Baseline Visit)

Cognitive Domain	Difference at baseline between the stereo acuity non-impaired and impaired	Annual rate of change for reference group (no stereoacuity impairment at baseline)	Difference (acceleration) in annual rate of change in the slope for group with stereoacuity impairment at baseline
	β (95% CI)	*β (95% CI)*	*β (95% CI)*
Language	-0.023 (-0.147 to 0.102)	**-0.040 (-0.045 to -0.034)**	**-0.020 (-0.034 to -0.005), 0.007**
Memory	-0.028 (-0.179 to 0.124)	**-0.029 (-0.036 to -0.022)**	**-0.032 (-0.052 to -0.013), 0.001**
Attention	-0.065 (-0.191 to 0.061)	**-0.065 (-0.074 to -0.057)**	-0.014 (-0.037 to 0.009), 0.23
Executive function	**-0.147 (-0.273 to -0.021)**	**-0.037 (-0.043 to -0.030)**	-0.005 (-0.022 to 0.011), 0.52
Visuospatial ability	-0.062 (-0.204 to 0.080)	**-0.046 (-0.055 to -0.037)**	-0.022 (-0.046 to 0.002), 0.07

All models adjusted for baseline stereo impairment status, age, sex, race, education, smoking status, hypertension, and diabetes
Bold: P<0.05

© 2021 Varadaraj V et al. *JAMA Network Open.*

eTable 4. Demographic and Clinical Characteristics of Participants at Baseline, Baltimore Longitudinal Study on Aging (2003-2019), by Inclusion in the Longitudinal Sensitivity Analysis

**Demographics**	**Only baseline visit N=188**	**Two or more visits N=1014**	**p-value[a]**
Age, in years, mean (SD) [range]	69.8 (8.4) [60-94]	71.3 (8.6) [60-93]	**0.026**
Age categories, in years, N (%)			0.07
60-79	157 (83.5)	788 (77.7)	
≥80	31 (16.5)	226 (22.3)	
Sex, N (%)			0.31
Female	89 (47.3)	521 (51.4)	
Male	99 (52.7)	493 (48.6)	
Race, N (%)			0.83
White	130 (69.1)	723 (71.3)	
Black	47 (25.0)	234 (23.1)	
Other	11 (5.9)	57 (5.6)	
Education, N (%)			0.94
High school or less	11 (5.9)	58 (5.7)	
At least some college, completed college	60 (31.9)	312 (30.8)	
Graduate school	117 (62.2)	644 (63.5)	
Number of visits, mean (SD)	NA	5.0 (2.4)	NA
Follow-up time, in years, mean (SD)	NA	8.1 (4.0)	NA
Number of visits, N (%)			
Two visits	NA	160 (15.8)	NA
≥Three visits	NA	854 (84.2)	
**Health Measures**			
Smoking, N (%)			0.48
Never/Quit >10 years ago	177 (95.2)	975 (96.3)	
Current/Quit <10 years	9 (4.8)	38 (3.7)	
Body Mass Index, in kg/m^2, N (%)			**0.03 (test for trend)**
<25	56 (29.9)	375 (37.0)	
25 to <30	77 (41.2)	409 (40.3)	
≥30	54 (28.9)	230 (22.7)	
Diabetes, N (%)	47 (25.0)	189 (18.6)	**0.04**
Hypertension, N (%)	80 (42.6)	361 (35.6)	0.07
**Visual Measures**			
Visual Acuity (logMAR), mean (SD)	0.16 (0.16)	0.16 (0.16)	0.96
Contrast Sensitivity[b] (log units), mean (SD)	1.91 (0.12)	1.91 (0.12)	0.89
Stereo Acuity Impairment (worse than 60 arcsec), N (%)[c]	25 (13.7)	136 (14.0)	0.91
Any Vision Impairment, N (%)[d]	53 (28.5)	268 (27.2)	0.71
**Cognitive Domain Scores**			
Language, mean (SD)	-0.020 (0.763)	0.019 (0.732)	0.50
Memory, mean (SD)	-0.186 (0.981)	0.014 (0.939)	**0.009**
Attention, mean (SD)	-0.080 (0.932)	0.003 (0.774)	0.25
Executive function, mean (SD)	-0.043 (0.929)	0.015 (0.801)	0.42
Visuospatial ability, mean (SD)	-0.132 (0.890)	-0.004 (0.864)	0.066

Abbreviations: logMAR=Logarithm of the Minimum Angle of Resolution, SD=standard deviation
[a]For *P* values, chi-squared test, test for trend, or *t* test was used as appropriate.
[b] Missing for 3 (only baseline) and 20 (two or more visits)
[c] Missing for 5 (only baseline) and 40 (two or more visits)
[d] Either visual acuity, contrast sensitivity, or stereo acuity impairment; missing for 2 (only baseline) and 28 (2 or more visits)

© 2021 Varadaraj V et al. *JAMA Network Open.*
